# Death of the Living Duplex Child

**Published:** 1829-12

**Authors:** 


### DEATH OF THE LIVING DUPLEX CHILD.

In our last Number we gave an account of a living duplex child: twin females, furnished with two heads, two necks, and four arms, but grafted or united side to side, so as to form only one female body, terminating in two legs, or inferior extremities, of usual shape. The death of these children was announced in the Journal du Commerce a few days ago. Since their departure from Italy, the health of Harriet gradually declined, and for some days there were no hopes of her recovery; while Christina had only a slight cold. Scarcely had Harriet ceased to breathe, when Christina refused the breast, and soon expired. The Academy of Sciences has procured a model of this remarkable *lusus naturæ;* and it is hoped that permission will be granted to embalm so curious a specimen of the vagaries of nature.

